# Galactosaminogalactan orchestrates *Verticillium dahliae* virulence and rhizosphere microbial ecology through multi-partite interactions

**DOI:** 10.1093/ismejo/wrag123

**Published:** 2026-05-17

**Authors:** Xueping Xu, Yingqing Tan, Sitong Xiecun, Junyao Wang, Wennan Zhao, Lei Wang, Renyu Dai, Liting Tang, Xianbi Li, Dan Jin, Yanhua Fan

**Affiliations:** College of Agronomy and Biotechnology, Southwest University, Chongqing 400715, China; College of Agronomy and Biotechnology, Southwest University, Chongqing 400715, China; College of Agronomy and Biotechnology, Southwest University, Chongqing 400715, China; College of Agronomy and Biotechnology, Southwest University, Chongqing 400715, China; College of Agronomy and Biotechnology, Southwest University, Chongqing 400715, China; College of Agronomy and Biotechnology, Southwest University, Chongqing 400715, China; College of Agronomy and Biotechnology, Southwest University, Chongqing 400715, China; College of Agronomy and Biotechnology, Southwest University, Chongqing 400715, China; College of Agronomy and Biotechnology, Southwest University, Chongqing 400715, China; College of Agronomy and Biotechnology, Southwest University, Chongqing 400715, China; College of Agronomy and Biotechnology, Southwest University, Chongqing 400715, China

**Keywords:** *V. dahliae*, GAG polysaccharides, cell wall, pathogenesis, microbiome, multi-functional molecule

## Abstract

The infection of plants by soil-borne fungal pathogens is a complex process and depends on their adhere ability to host surfaces and interactions with the rhizosphere bacteria. In this study, we identify galactosaminogalactan (GAG) as a pivotal virulence determinant in the pathogenesis of *Verticillium dahliae*. By characterizing the *VdGAG* biosynthetic gene cluster, we show that the glycosyltransferase VdGtb is essential for GAG synthesis. The knockout of GAG increased fungal sensitivity to the cell wall-perturbing agent calcofluor white and reduced mycelial ball formation in liquid culture. The absence of GAG polysaccharides reduced root-binding capacity by 50% and increased cotton immune responses 1 day post fungal infection. The *ΔVdGtb* mutant exhibited a significant 30.5% decrease in the pathogenicity toward cotton seedlings compared with the wild type V991. Microbiome and bacterial enrichment analysis indicate that the GAG polysaccharides promote the enrichment of soil bacteria and alter the bacterial community structure in the plant rhizosphere. Several bacteria enriched by GAG-contained fungal cells, including *Achromobacter animicus*, *Pseudomonas aeruginosa*, and *Acinetobacter pittii* exhibited strong growth-inhibitory effects against *V. dahliae* and showed distinct effects on fungal virulence in a GAG-dependent manner. Together, these results reveal that GAG is not merely a cell wall component but a multi-functional molecule that orchestrates fungal protection, host infection, and inter-kingdom microbial communication.

## Graphical Abstract

**Graphical Abstract:**
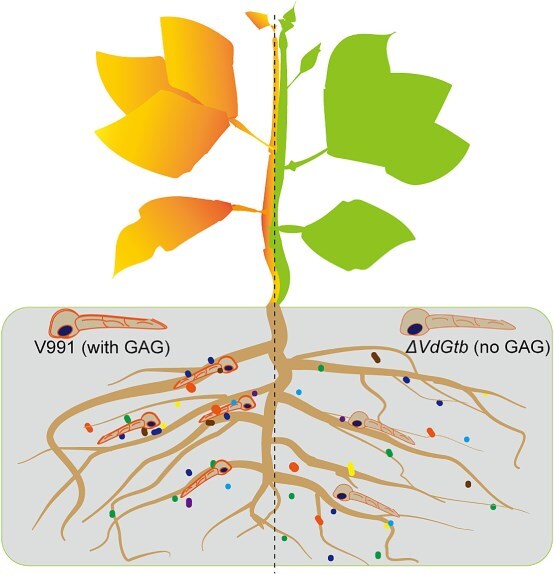

## Introduction

Galactosaminogalactan (GAG) is a key component of the fungal extracellular matrix, acting as an adhesive and modulating host immunity for fungal cells [1, 2]. It was first isolated from the cell wall of *Aspergillus fumigatus* and has been shown to play important roles in mycelial aggregation and fungal adhesion to host surfaces [3, 4]. The biosynthesis of GAG polysaccharides in *Aspergillus. niger* involves five clustered genes, which encode uridine diphosphatase–glucose-4-epimerase (Uge), glycoside hydrolase (Sph, Ega), deacetylase (Agd), and glycosyltransferase (Gtb), respectively [5, 6]. Uge converts the substrates UDP-glucose and UDP-N-acetylglucosamine into UDP-galactose and UDP-N-acetylgalactosamine, respectively. Gtb then linearly polymerizes these two products to form a polysaccharide that is subsequently transported to the extracellular space. The GAG polymer is partially deacetylated by Agd, thereby rendering it cationic, which facilitates the adhesion of GAG polysaccharides to the mycelial surface. Deletion of *Agd3* and *Uge3* in *A. fumigatus* significantly impairs fungal adhesion and biofilm formation [7]. In the insect-pathogenic fungus *Metarhizium robertsii*, GAG polysaccharides mediate the formation of appressorial mucus, a structure essential for infective differentiation [8]. There is also evidence that GAG polysaccharide biosynthesis gene clusters are conserved across at least 28 fungal species, primarily within the *Ascomycota* and *Basidiomycota* phyla [7]. Together, these findings indicate that GAG polysaccharides are widely distributed in fungi and play important roles in environmental adaptation and host interactions.

*Verticillium dahliae* is a soil-borne vascular plant pathogen that can cause diseases in ˃600 dicotyledonous plant species, including cotton, tomatoes, peppers, and potatoes [9, 10]. Infection by *V. dahliae* leads to leaf yellowing, wilting, vascular discoloration, ultimately plant death, causing severe yield losses. To date, no effective biological control strategies are available for this pathogen [11, 12]. In soil, the hyphae of *V. dahliae* grow toward the host root surface, or microsclerotia and conidia adhere to host roots and germinate, forming infection structures that penetrate epidermal cells and colonize host tissues [13]. Therefore, early adhesion and colonization in the host rhizosphere represent critical steps in *V. dahliae* pathogenesis. For example, deletion of the *Vta2* and *Vta3* genes, encoding key transcription factors for fungal infection of host plants, weakens fungal adhesion to host roots and significantly reduces virulence [14, 15]. Similarly, deletion of *VdMsb* (a transmembrane mucin-encoding gene) reduced root colonization and attenuated fungal virulence in *Arabidopsis thaliana* [16].

Numerous studies have shown that the homeostasis of rhizosphere communities is disrupted during fungal pathogen invasion, which in turn influences disease development. For instance, during infection of tomato seedlings by *Fusarium oxysporum*, the diversity of rhizosphere fungi and bacteria is significantly reduced compared with healthy plants, suggesting a negative correlation between microbial diversity and pathogen abundance [17]. *Funneliformis mosseae* can attract *Pseudomonas putida* KT2440 by secreting the signaling molecule cysteine, thereby promoting bacterial colonization in the soybean rhizosphere [18]. *V. dahliae* secretes the antibacterial effector VdAve1, which reshapes the soil microbial community and promotes fungal colonization of tomato and cotton [19]. From the plant perspective, hosts also actively manipulate rhizosphere microbial communities to enhance disease resistance. For example, when wheat is infected with *F. pseudograminearum*, the diazotrophic rhizobacterium *Azospirillum diazotrophicum* SR80 is significantly enriched, promoting plant growth and inducing strong disease resistance [20]. Studies have also shown that root-leaked glutamine guides bacterial colonization, promoting the formation of beneficial biofilms at specific root sites that protect plants against external damage [21]. Plants can also sense bacterial exotoxins to distinguish between beneficial and pathogenic microbes, thereby activating distinct immune responses [22]. Collectively, these studies indicate that both plants and fungi can actively reshape rhizosphere microbial communities to favor their own survival.

Although the changes in the rhizosphere microbial homeostasis have been extensively observed, the molecular mechanisms causing these changes remain largely unknown in most cases. Interactions among fungi, plants, and soil bacteria are particularly complex, and identifying the key molecules or structures that mediate these interactions is crucial. In this study, we demonstrate that loss of GAG in *V. dahliae* significantly impairs the fungal aggregation and host adhesion, thus reducing pathogenicity. GAG promotes bacterial enrichment in the rhizosphere and reshapes the rhizosphere bacterial community. Bacteria enriched by GAG exhibit diverse activities, including inhibition of fungal growth and suppression of plant disease. These results reveal GAG play multiple functions in *V. dahliae*, including protecting fungal cells, mediating fungus-plant interactions, and serving as a platform for fungus-bacterium interactions.

## Materials and methods

### Strain culture and vector construction

The wild-type *V. dahliae* strain V991 and its derived mutants were cultured at 26°C in liquid or solid (1.5% agar) media, including Czapek-Dox medium (CZM; BD, USA), potato dextrose broth (PDB; BD, USA), and Sabouraud dextrose broth (SDB; containing 10 g glucose, 2.5 g peptone, and 5 g yeast extract per liter). *Escherichia coli* and soil bacteria were grown on LB medium (10 g tryptone, 10 g NaCl, 5 g yeast extract per liter), and *Agrobacterium tumefaciens* (strain AGL-1) was grown on YEB medium (10 g tryptone, 1 g yeast extract, 5 g sucrose, and 0.5 g MgSO₄·7H₂O per liter).

The *ΔVdGtb* knockout mutant was generated via homologous recombination using the pK2-HygB vector and confirmed by real-time quantitative polymerase chain reaction (RT-qPCR). For genetic complementation, an ~11.7 kb genomic DNA fragment containing the native *VdGtb* promoter and coding sequence was cloned into pK2 vector carrying a *Geneticin* resistance gene. Fungal transformation was performed as previously described [23]. All primers used in this study were listed in Supplementary Table S2.

### Bioinformatics analysis

The *VdGtb* gene sequence (NCBI accession number: VDAG_08041) was obtained from the NCBI database. Protein domain prediction was performed using the SMART online tool [24]. Phylogenetic analysis was conducted using *MEGA 11.0* with the Neighbor-Joining method [25]. The accession numbers of the homologous proteins used to construct the phylogenetic tree are provided in Supplementary Text S1.

### Scanning electron microscopy

Fungal conidial suspensions were prepared using 0.05% Tween-80 from 6-day-old potato dextrose agar (PDA) cultures. Conidia were collected by centrifugation at 4500 rpm for 3 min and washed three times with 0.01 M PBS (pH 7.0). Samples were fixed overnight at 4°C in 4% paraformaldehyde, washed three times with PBS (15 min each), and dehydrated sequentially through a graded ethanol series (30%–95%, 15 min each), followed by 100% ethanol for 20 min. After a final centrifugation, conidia were air-dried and observed using a scanning electron microscope (Phenom XL G2, Netherlands).

### Lectin staining of *V. dahliae*

*V. dahliae* mycelial cultures grown in PDB for 15 h were collected and resuspended in 0.01 M PBS (pH 7.0). Soybean lectin dye (Vector, Germany) was added to a final concentration of 20 μg / mL. Staining was performed according to the manufacturer’s protocol. Fluorescence signals were detected using a Leica SP8 confocal microscope with an excitation wavelength of 488 nm and an emission range of 495–535 nm. Conidia were collected from 7-day PDA plates and resuspended in 0.01 M PBS (pH 7.0). Conidial soybean agglutinin staining was performed as for hyphae.

### Polysaccharides extraction and high-performance liquid chromatography detection

Crude polysaccharides were isolated from *V. dahliae* cultures grown in SDB for 5 days using ethanol precipitation as previously described [26]. The concentration of crude polysaccharides was quantified by the phenol-sulfuric acid method [27]. The monosaccharide composition of GAG polysaccharides was determined by high-performance liquid chromatography (HPLC) following hydrolysis, reduction, and derivatization [28]. HPLC analysis was performed with an Agilent Zorbax SB-C18 column (250 mm × 4.6 mm, 5 μm). The mobile phase consisted of 0.05 M phosphate buffer (pH 7.0, phase A) and acetonitrile (phase B). Isocratic elution was carried out at 85% A and 15% B with a flow rate of 1.0 mL / min. Eluted compounds were detected using a UV detector at 250 nm. Galactose and N-acetylgalactosamine dissolved in water were used as standards.

### Mutant growth phenotypes and stress tolerance

Fungal conidial suspension (100 μL, 1 × 10^7^ conidia / mL) was inoculated into 50 mL of liquid media, including CZM, PDB, and SDB. Cultures were incubated at 26°C with shaking at 200 rpm for 3 days, after which growth phenotypes were observed. Penetration assays were performed by inoculating 3 μL of conidial suspension (1 × 10^7^ conidia / mL) onto sterile cellophane membranes overlaid on CZA medium. Plates were cultured at 26°C for 3 days, after which the cellophane was removed. Fungi were grown for an additional 3 days to form colonies. Biofilm formation was assayed by inoculating 25 μL of conidial suspension (1 × 10^7^ conidia / mL) into 400 μL CZM medium in 12-well plates and incubated at 26°C for 20 h. After incubation, the medium was carefully removed using a pipette. Excess stain was removed and the wells were gently washed twice with sterile water. Subsequently, 100 μL of 1% crystal violet solution was added to each well and incubated for 10 min at room temperature. The wells were then washed twice with sterile water. For quantification, the bound crystal violet was solubilized with 125 μL of anhydrous ethanol and the absorbance was measured at 600 nm. Conidiation, conidial germination, and fungal resistance to abiotic stresses were assessed following previous methods [29, 30]. The procedures for analyzing the relative expression levels of the *VdGAG* gene cluster under different culture conditions are detailed in Supplementary Text S2.

### Fungal pathogenicity

Fungal virulence was evaluated with 14-day-old cotton seedlings and disease severity was assessed as previously described [29, 31]. For each fungal strain, 30 plants were treated. All plants were maintained in a growth chamber at 25–27°C with regular watering. To detect the expression of immune-related genes, root tissues were harvested at 1, 2, and 3 days post-inoculation (dpi) and total RNA was extracted. RT-qPCR was performed using gene-specific primers with Bio-Rad CFX Manager 3.0 software. Gene expression levels were normalized to the *GAPDH* gene.

To assess fungal virulence against *A. thaliana*, the roots of sterile 10-day-old seedlings were inoculated with 5 μL of conidial suspension (5 × 10^6^ conidia / mL). The inoculated plants were cultured in a growth incubator at 26°C under a 16 h light / 8 h dark photoperiod. Disease symptoms were evaluated at 10 days post-inoculation and disease severity was graded on a 0–4 scale: 0 (no symptoms), 1 (<25% leaf area affected), 2 (25–50% affected), 3 (50–75% affected), and 4 (>75% affected). To evaluate fungal adhesion toward plant roots, *A. thaliana* inoculated with fungi for 15 h were collected and rinsed twice with sterile water. The roots were gently ground with 500 μL of sterile water in a mortar. The resulting homogenate was serially diluted (10-fold) and 100 μL was spread onto CZA plates. Plates were incubated at 26°C for 3 days and fungal colonies were counted to determine colony-forming units (CFU).

### Microbiome analysis

To assess the impact of GAG on cotton rhizosphere microbial communities, 20-day-old cotton seedlings were inoculated with a conidial suspension (4.5 mL per plant, 2 × 10^7^ conidia / mL) and cultivated in a growth chamber at 26°C with a photoperiod of 16 h light / 8 h dark. After 10 days, rhizosphere soil was collected from each plant for microbiome sequencing analysis [32]. Control plants were inoculated with an equivalent volume of sterile water. Each treatment group consisted of six independent biological replicates. Detailed microbiome data acquisition and analysis are described in Supplementary Text S3.

### Interactions between soil bacteria and *V. dahliae*

To identify bacteria enriched by *V. dahliae* in the cotton rhizosphere, 20 g of rhizosphere soil was mixed with 80 mL of sterile water, and incubated at 37°C with shaking (200 rpm) for 5 h. The mixture was filtered through one layer of filter paper. Subsequently, 30 mL of the resulting soil filtrate was co-incubated with *V. dahliae* hyphae (pre-grown in 100 mL PDB for 5 days) at 26°C with shaking (200 rpm) for 12 h to allow bacterial attachment. Hyphae with attached bacteria were harvested by filtration through three layers of filter paper and rinsed twice with sterile water. The obtained hyphae were resuspended in sterile water and got sonicated (120 W for 10 min) to detach bacteria. After filtration, the bacterial suspension was inoculated onto LB agar plates and incubated until colonies formed. The bacterial 16S rRNA gene was amplified using the universal primer pair 27F and 1492R [33]. Detailed experimental procedures for bacterial identification and bacterial-fungal interactions are provided in Supplementary Text S4.

### Effects of bacteria on the virulence of various *V. dahlia*e strains

*V. dahliae* conidial suspensions (6 × 10^7^ conidia / mL) and bacterial suspensions (OD_600_ = 0.02 or 0.2) were mixed at a 1:1 ratio. Fourteen-day-old cotton seedlings were treated by drenching 10 mL of the mixed fungal-bacterial suspension to the root zone of each plant. All plants were maintained at 25°C under a 16 h light / 8 h dark photoperiod. For each fungal strain, 30 plants were treated. Disease symptoms were evaluated 25 days post-inoculation.

### Data analysis

Data analyses were carried out on *GraphPad Prism* (version 8) and *SPSS* (version 26) software. All data were tested for normality using the Shapiro–Wilk test. For comparisons between two groups, normally distributed data were analyzed by unpaired *t-*test. For comparisons among multiple groups, normally distributed data were analyzed by one-way ANOVA followed by Tukey’s multiple comparisons test, whereas non-normally distributed data were analyzed by the Kruskal–Wallis test followed by Dunn’s multiple comparisons test. Effect sizes are reported as the correlation coefficient *r*, |*r*| ≥ 0.5, 0.3–0.5, and < 0.3 represent large, medium, and small effects, respectively. The corresponding precise *r* and *P*-values ​​are marked within the figure.

## Results

### *V. dahliae* synthesizes GAG polysaccharides

**Figure 1:**
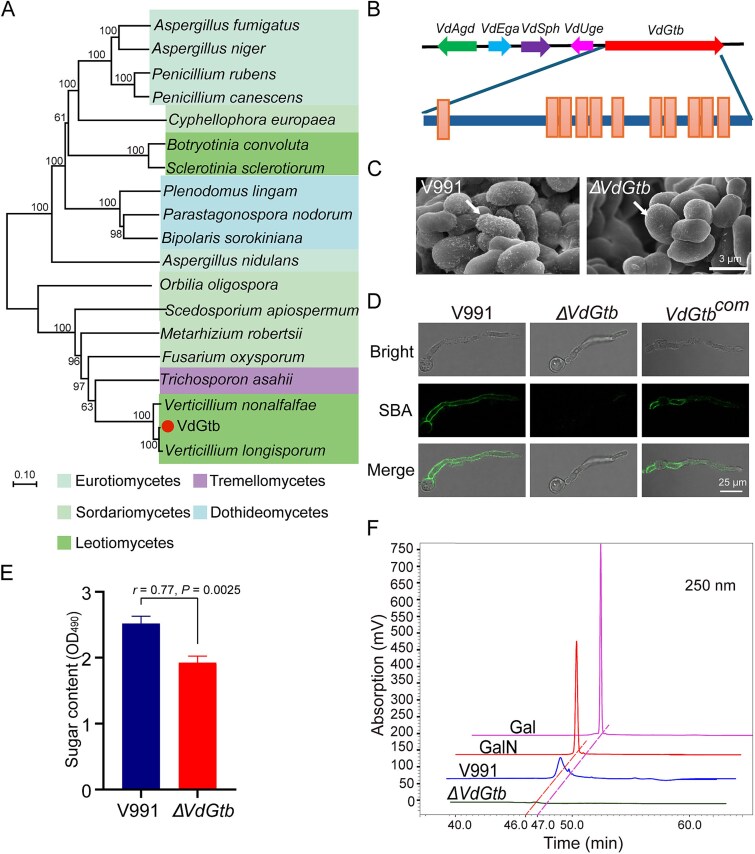
*V. dahliae* synthesizes GAG polysaccharides. (A) The phylogenetic tree of VdGtb protein. The sequences of VdGtb and its homologous proteins from other filamentous fungi were obtained from NCBI and the tree was constructed using the neighbor-joining method. Bar, 0.10 substitutions per site. (B) Schematic representation of the GAG synthesis gene cluster and the domain architecture of VdGtb. Five genes were identified in the GAG cluster, including uridine diphosphatase-glucose-4-epimerase (Uge), glycoside hydrolase (Sph, Ega), deacetylase (Agd), and glycosyltransferase (Gtb). Eleven transmembrane regions were identified in VdGtb. (C) Scanning electron microscopy images of conidia from the wild-type (V991) and *VdGtb* knockout (*ΔVdGtb*) strains. Scale bar, 3 μm. (D) Soybean agglutinin staining of GAG polysaccharides. Scale bar, 25 μm. (E) Quantification of secreted polysaccharides in culture filtrates. The vertical axis represents the absorbance at 490 nm, which serves as an indicator of crude sugar content in the samples. Plotted data are represented as the mean ± SEM. Data were normally distributed (Shapiro–Wilk test, *P* > .05) and analyzed using an unpaired *t-*test. n ≥ 3. (F) High-performance liquid chromatography analysis of galactose (Gal, retention time: 47.0 min) and galactosamine (GalN, retention time: 46.1 min) contents in polysaccharides from culture filtrates.

Using GAG biosynthetic gene cluster sequences from *A. fumigatus* as query sequences, we identified a conserved GAG synthesis gene cluster (named *VdGAG*) in the *V. dahliae* genome, including *VdAgd*, *VdEga*, *VdSph*, *VdUge*, and *VdGtb*. Among them, *VdGtb* encodes a glycosyltransferase consisting of 2856 amino acids. Phylogenetic tree analysis showed that VdGtb was closely related to the glycosyltransferase proteins in Ascomycetes and Basidiomycetes (Fig. 1A, Table S1). Protein structure prediction indicated that VdGtb had 11 transmembrane domains (Fig. 1B). The expression of the *VdGAG* gene cluster was detected in both the hyphae and conidia of *V. dahliae*, however, most of the genes (except *VdSph*) showed higher expression levels in conidia (Fig. S1A). Using homologous recombination and *Agrobacterium*-mediated genetic transformation methods, we obtained *VdGtb* knockout and complementation strains in *V. dahliae* (Fig. S2). Scanning electron microscopy and soybean lectin (a GAG-specific fluorescent dye) staining showed that the *ΔVdGtb* mutant lacked extracellular GAG polysaccharides in both hyphae and conidia (Fig. 1C and D;  Fig.  S1B). Consistently, the total sugar content and GAG components, including galactose (Gal) and N-acetylgalactosamine (GalN), in the culture medium of *ΔVdGtb* were significantly lower than those of the wild-type strain V991 (Fig. 1E and F). These results demonstrate that *V. dahliae* is able to synthesize GAG polysaccharides and the loss of the glycosyltransferase gene *VdGtb* leads to defective GAG biosynthesis.

### Lack of GAG decreases fungal resistance to cell wall stress and affects mycelial aggregation

**Figure 2:**
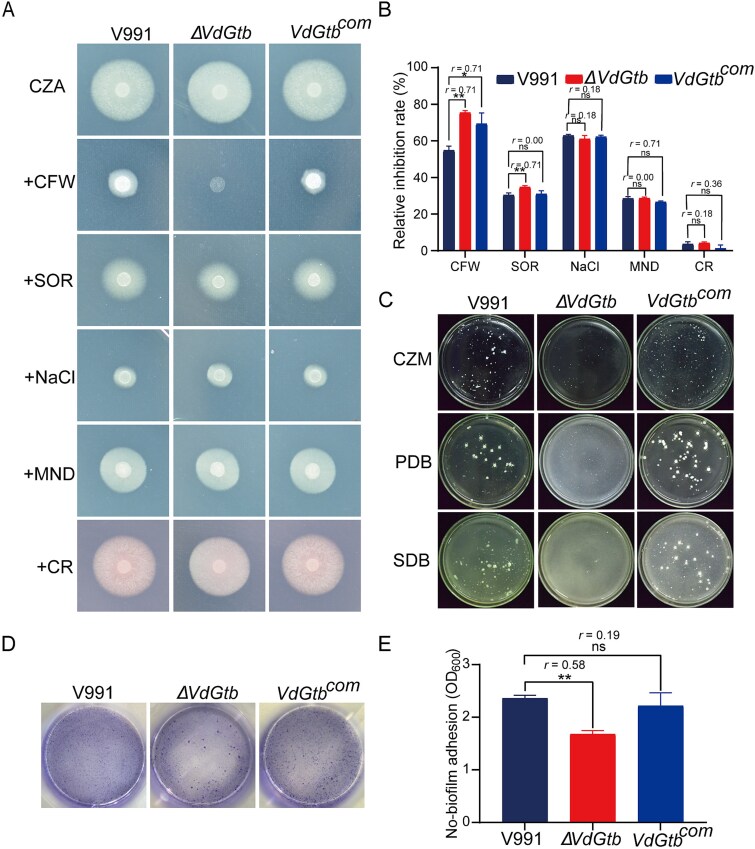
Effects of GAG polysaccharides on hyphal aggregation, adhesion, and abiotic stress tolerance. (A) Abiotic stresses tolerance analysis of various strains. Fungal strains were inoculated on CZA media containing CFW, Sorbitol (SOR), NaCl, Menadione (MND), and Congo Red (CR), respectively. (B) Relative growth inhibition rates of the fungal strains shown in (A). (C) Hyphal aggregation in liquid cultures. Fungal strains were cultured in CZM, PDB, and SDB for 5 days. (D) Non-biofilm adhesion to abiotic surfaces assessed by crystal violet staining. (E) Quantification of non-biofilm adhesion to abiotic surfaces (D) via OD_600_ measurement. Plotted data in B and E are represented as the mean ± SEM. Data were normally distributed (Shapiro–Wilk test, *P* > .05) and analyzed using one-way ANOVA followed by Tukey’s multiple comparisons test. n ≥ 3. Significant differences are denoted by asterisks: ^*^P < .05, ^**^P < .01; ns, not significant. *r*, effect size.

Lack of GAG did not cause significant changes in fungal colony growth, conidial germination, or penetration ability (Fig. S3). However, loss of GAG polysaccharides significantly reduced fungal resistance to cell wall disturbing agent calcofluor white (CFW), resulting in a 75.4% relative growth inhibition rate, which was significantly higher than that of the wild-type strain (54.8%) (Fig. 2A and B). In liquid cultures, *V. dahliae* hyphae aggregate to form hyphal balls. In contrast, the *ΔVdGtb* mutant failed to form aggregated hyphal balls in all three tested liquid media (CZM, PDB, and SDB), and instead exhibited dispersed, flocculent hyphae (Fig. 2C). In addition, the biofilm-forming ability of *V. dahliae* was significantly impaired in the *ΔVdGtb* mutant (Fig. 2D and E).

### GAG polysaccharides mediate fungal interactions with cotton plants

**Figure 3:**
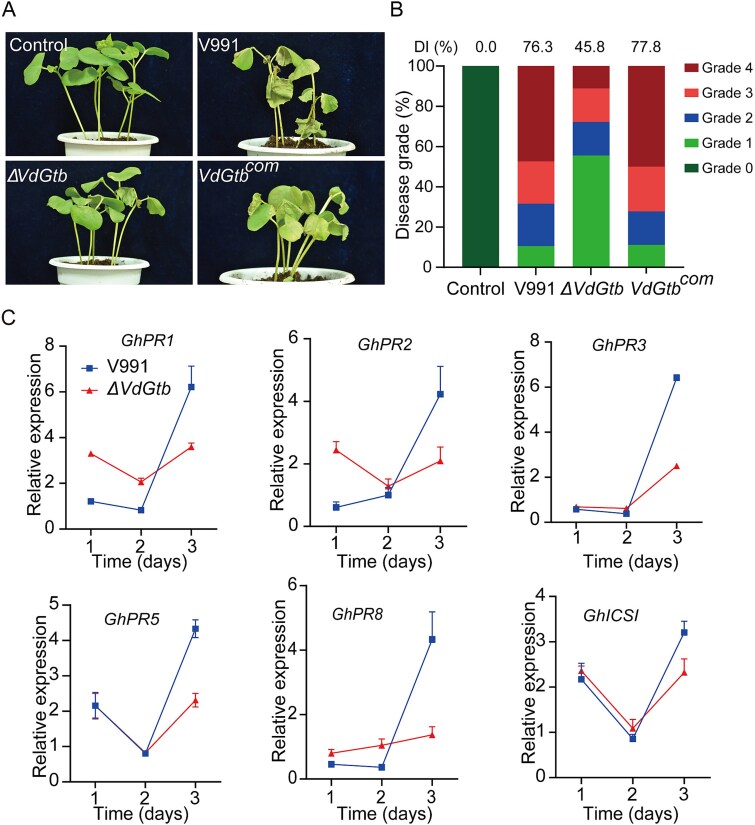
GAG polysaccharides are required for full pathogenicity of *V. dahliae* on cotton. (A) Disease symptoms on cottons inoculated with various fungal strains. (B) Disease index and distribution of disease grades corresponding to (A). (C) Relative expression of cotton immune-related genes in response to infection by V991 or *ΔVdGtb*.

To explore the functions of GAG polysaccharides in fungal pathogenicity, cotton seedlings were inoculated with the tested strains using the root-dip method. Cotton plants inoculated with the wild-type and complemented strains showed severe leaf yellowing and wilting symptoms at 14 days post inoculation, whereas plants infected with *ΔVdGtb* displayed milder symptoms (Fig. 3A). The disease index of cotton seedlings inoculated with *ΔVdGtb* was 45.8%, which was significantly lower than that of the wild-type and complemented strains (76.3% and 77.8%, respectively) (Fig. 3B). Reduced virulence of *ΔVdGtb* was also observed in *A. thaliana* (Fig. S4A and B*)*. Fungal adhesion to host roots was further evaluated using *A. thaliana*. The number of wild-type hyphae adhering to roots was about two-fold higher than that of *ΔVdGtb* (Fig. S5A and B), indicating that GAG deficiency significantly decreases fungal adhesion to host roots. We further examined the expression levels of several cotton defense-related genes, including *GhPR1*, *GhPR2*, *GhPR3*, *GhPR5*, *GhPR8*, and *GhICS1.* Compared with V991-infected seedlings, *GhPR1* and *GhPR2* exhibited higher expression levels at 1 day post inoculation in *ΔVdGtb*-infected plants, whereas the other four genes showed comparable levels among various treatments. At 3 days post inoculation, all tested genes showed higher expression levels in V991-infected seedlings than in *ΔVdGtb-*infected seedlings (Fig. 3C).

### GAG polysaccharides affect the composition of rhizosphere soil microbial

**Figure 4:**
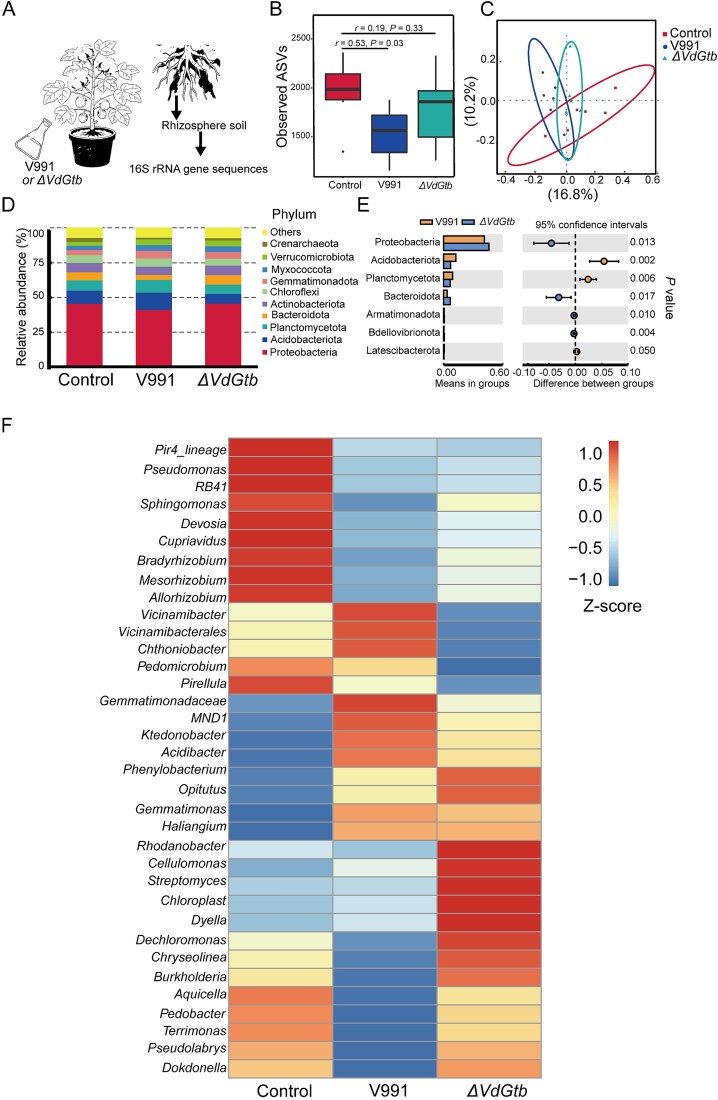
GAG polysaccharides alter the bacterial community in the cotton rhizosphere. (A) Schematic of the experimental design for rhizosphere microbiome analysis. (B) Bacterial α-diversity (Observed ASVs) in cotton rhizosphere inoculated with V991 and *ΔVdGtb* (Kruskal–Wallis test, n = 6). (C) Principal coordinate analysis (PCoA) of bacterial community composition based on 16S rRNA gene sequencing. (D) Relative abundance of the top 10 bacterial phyla. (E) Phyla showing significantly different relative abundance between V991 and *ΔVdGtb* treatments (Student’s *t-*test, n = 6). (F) Heatmap showing the clustering of rhizosphere bacterial communities at the genus level. The color scale from −1 to 1 represents the Z-score normalized relative abundance of each bacterial genus. Shade intensity reflects deviations from the average relative abundance across all samples.

As a soil-borne pathogenic fungus, *V. dahliae* maintains an extensive interaction network in the complex soil environment. We therefore explored that GAG polysaccharides function in the interaction between *V. dahliae* and the host rhizosphere microorganisms. Wild type V991 and *ΔVdGtb* strains were applied to the cotton rhizosphere and bacterial community composition was analyzed by 16S rRNA gene sequencing (Fig. 4A). All samples achieved Good’s coverage values over 98%, indicating that our sequencing depth was sufficient to reflect the vast majority of microbial information in the samples (Fig. S6A). After inoculation with V991, the observed amplicon sequence variants (ASVs) of cotton rhizosphere soil were significantly lower than those of untreated soils (Fig. 4B). In contrast, *ΔVdGtb* treatment did not result in significant changes in the Shannon index or Simpson index compared to the control group (Fig. S6B and C). β-diversity was assessed using the Bray–Curtis dissimilarity matrix and a PCoA ordination. PCoA1 and PCoA2 explained 16.8% and 10.2% of the total variation, respectively. Samples from different treatment groups showed a certain degree of separation in the PCoA plot (Fig. 4C), indicating that V991 significantly altered the rhizosphere microbial community structure and this effect was attenuated upon GAG deficiency. At the phylum taxonomic level, significant differences in the bacterial community composition were observed between V991 and *ΔVdGtb* treatments. *Proteobacteria* were the dominant phylum in the cotton rhizosphere soil before and after fungal inoculation. After V991 treatment, the relative abundance of *Proteobacteria* was 41.5%, ~5% lower than that in *ΔVdGtb*-treated and control soils. Infection with V991 significantly increased the relative abundance of Acidobacteriota, Planctomycetota, Gemmatimonadota, Myxococcota, and Verrucomicrobiota compared to the control and the *ΔVdGtb* groups (Fig. 4D and E). At the genus level, bacteria including *Gemmatimonas*, *Ktedonobacter*, *Phenylobacterium*, were significantly enriched in the presence of GAG polysaccharides during infection, whereas in the *ΔVdGtb* mutant, these genera exhibited opposite pattern (Fig. 4F).

### GAG polysaccharides serves as the platform for interactions between *V. dahliae* and bacteria

**Figure 5:**
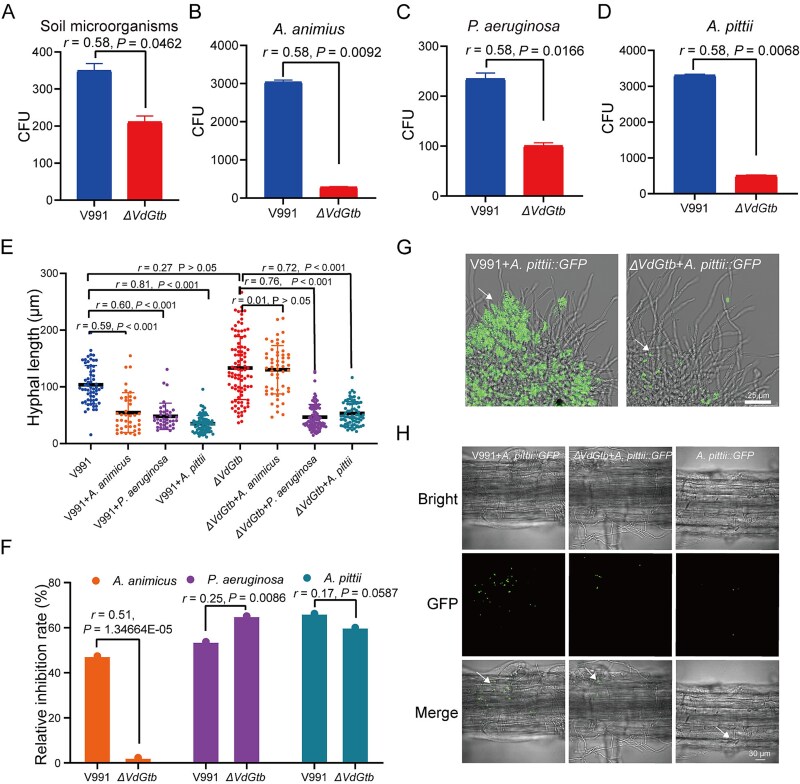
GAG polysaccharides mediate the interactions between *V. dahliae* and specific soil bacteria. (A–D) Culturable bacterial colonies recovered from fungal hyphae after co-culture with soil microorganisms (A), *A. animicus* (B), *P. aeruginosa* (C), and *A. pittii* (D). (E) Hyphal length of V991 and *ΔVdGtb* in PDB after co-culture with the indicated bacteria. (F) Relative inhibition of hyphal elongation in the liquid co-culture assay shown in (E). (G) Microscopic observation of *A. pittii::GFP* adhesion to V991 and *ΔVdGtb* hyphae. Scale bars, 25 μm. (H) Microscopic observation of fungus-bacterium attachment to *Arabidopsis* roots. Scale bar, 50 μm and 25 μm. Data in (E) and (F) are presented as mean ± SEM. Data were analyzed using the Kruskal-Wallis test followed by Dunn’s multiple comparisons test. Differences were considered statistically significant at *P* < .05. n ≥ 3.

The bacteria-enrichment ability of GAG was evaluated by co-culturing V991 and *ΔVdGtb* mycelia with soil bacterial suspensions. The results showed that loss of GAG polysaccharides significantly decreased the bacterial enrichment ability compared to the wild-type strain, resulting in a 1.7-fold decrease in the attached bacterial abundance (Figs 5A and S7A). Three bacterial strains, *Acinetobacter pittii, Achromobacter animicus*, and *Pseudomonas aeruginosa*, were isolated and selected for further analysis. The *ΔVdGtb* strain interacted weakly with all three bacteria compared with the wild-type strain, showing a 1.9-to 10.6-fold reduction in bacterial amounts (Figs 5B–D and S7B–D). The effects of these bacteria on *V. dahliae* growth were assessed by co-cultivation in liquid media or colony confrontation on solid media. In liquid co - culture assays, all three bacteria strongly inhibited the wild type with relative inhibition rates ranging from 53.7% to 66.3%. Treatment with *P. aeruginosa* resulted in a higher inhibition rate in *ΔVdGtb* than in the wild-type strain (65.1% vs. 53.7%), whereas co-culture with *A. animicus* had no significant effect on *ΔVdGtb* growth (Fig. 5E and F). In non-contact colony confrontation assays, only *P. aeruginosa* showed a stronger inhibitory effect on the wild-type strain than on *ΔVdGtb*, whereas the other two bacteria exhibited only weak inhibition of both strains (Fig. S7E and F). To further investigate the functions of GAG polysaccharides in fungus-bacterium interactions*,* the *GFP* gene with a constitutive promoter was introduced into *A. pittii*. The GFP-expressing *A. pittii* strain was co-cultured with the wild type and *ΔVdGtb* hyphae. The fluorescent signal analysis revealed a higher abundance of bacteria enriched on wild-type hyphae than on *ΔVdGtb* (Fig. 5G). In addition, co-inoculation of GFP-expressing *A. pittii* with fungal conidia onto *Arabidopsis* roots showed that fewer *A. pittii::GFP* cells were present on roots treated with *ΔVdGtb* compared with those infected with wild type (Fig. 5H).

**Figure 6:**
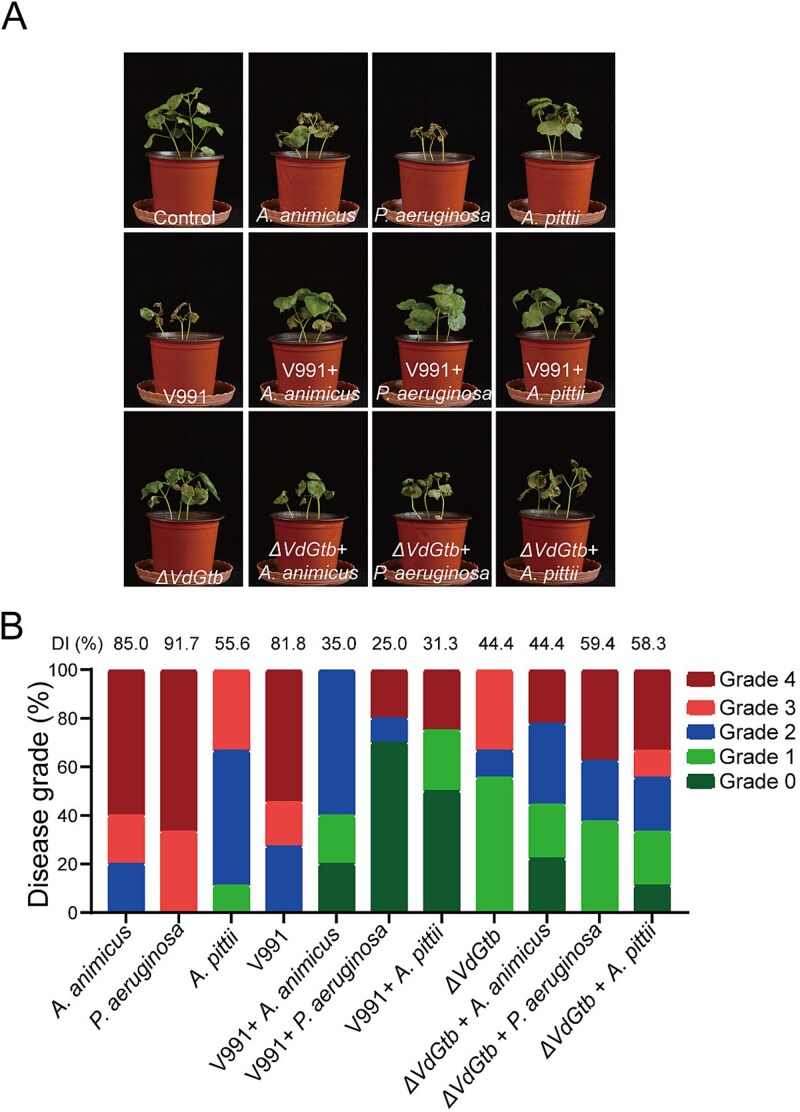
Effects of fungus-bacterium interactions on the virulence of *V. dahliae*. (A) Disease symptoms on cotton plants inoculated with V991 or *ΔVdGtb* alone or in combination with bacteria. The three bacteria, including *A. animicus*, *P. aeruginosa*, and *A. pittii*, were used at a concentration of OD_600_ = 0.1 and the disease symptoms were observed after 25 days. (B) Disease index and grade distribution corresponding to the experiment shown in (A). n ≥ 3.

The presence of both natural soil and bacteria increased the relative expression level of the *VdGAG* gene cluster to some extent (Fig. S8). Therefore, the effects of bacteria on fungal virulence were evaluated by co-inoculating bacteria with fungal conidia on cotton seedlings. At a high bacterial concentration (OD_600_ = 0.1), these bacteria alone induced severe disease symptoms (DI = 55.6 ~ 91.7). Although both bacteria and V991 individually caused disease in cotton, co-inoculation significantly alleviated disease symptoms, resulting in a lower DI (~30%) compared to either bacteria or the fungus alone (DI = 81.8)*.* Co-inoculation of the *ΔVdGtb* mutant with bacteria also reduced the disease symptoms relative to bacteria alone, although the protective effect was weaker than that observed in the wild type (Fig. 6)*.* At a low bacterial concentration (OD_600_ = 0.01), the bacteria alone did not induce obvious disease symptoms. Similarly, co-inoculation of *V. dahliae* with either *A. animicus* or *P. aeruginosa* reduced disease symptoms*.* Co-inoculation with *A. pittii* increased fungal virulence, resulting in a 21% increase in DI (45.7 vs 66.7) compared to *V. dahliae* alone. A similar trend was observed in the *ΔVdGtb* mutant, although the magnitude of the effects was weaker than that observed in the wild-type strain (Fig. S9).

## Discussion

The infection of hosts by plant pathogenic fungi is a complex process and adhesion ability plays a key role in this process [34]. Fungi adhere to plant outer surface mainly through water-insoluble extracellular matrices, including lipid-or polysaccharide-containing glycoproteins and polysaccharides [35, 36]. Our study presents evidence that a conserved GAG synthesis gene cluster in the genome of *V. dahliae* is responsible for GAG polysaccharide synthesis. Loss of the key glycosyltransferase gene *VdGtb*, which is required for GAG synthesis, reduces fungal biofilm formation and compromises fungal resistance to cell wall-perturbing stresses. In addition, GAG plays important roles not only in the interaction between *V. dahliae* and plant hosts but also as a platform for interaction between the fungus and bacteria, indicating its multiple functions in complex environmental conditions.

Loss of GAG decreased the infectious ability of *V. dahliae* against *A. thaliana* and cotton. The similar phenomenon has been observed in entomopathogenic fungus *Metarhizium robertsii* [8] and the opportunistic human fungal pathogen *Aspergillus* [37]. The impaired virulence possibly resulted from two aspects, decreased adhesion of the fungus to plant tissues and compromised immune reactions. Our results indicated *ΔVdGtb* strain has a weak binding ability to plant root compared to the wild type. When plant pathogenic fungi invade host plants, physical contact with cell walls or extracellular matrix components can directly induce host immune-related defense responses [38, 39]. In this process, a large number of immune-related genes in plant tissues, such as genes in salicylic acid, jasmonic acid, and other related pathways, were significantly up-regulated [40, 41]. In this study, we found that the loss of GAG polysaccharides in *V. dahliae* induced higher expression levels of *GhPR1* and *GhPR2* at the early infection stage (1 d post inoculation) in cotton seedlings, however all the tested immune-related genes showed lower expression levels compared to the wild-type strain at 3 d post infection. It has been reported that GAG polysaccharides have the molecular characteristics of masking pathogen associated molecular patterns (PAMPs) thus is able to decrease host immune reactions [3, 42]. We speculate that the lack of GAG polysaccharides leads to greater exposure of fungal PAMPs (such as glucan and chitin) present in the cell wall of *V. dahliae* to the plant, thereby eliciting a quicker and stronger plant immune response at the early stage of infection. At the same time, we do not rule out the possibility that GAG polysaccharides themselves may trigger the host’s immune response. With the development of fungal disease, wild type proliferated more quickly and induced stronger immune reactions compared to the *VdGtb* mutant at 3 d post inocualtion. The detailed mechanism of GAG polysaccharides involved in host plant immune reactions needs to be further studied in the future.

GAG polysaccharides, as an extracellular matrix component, are the "physical platform" for fungi to contact other microorganisms. We found that the application of wild-type *V. dahliae* or *ΔVdGtb* caused a decrease in the diversity and abundance of rhizosphere soil bacterial communities. However, *ΔVdGtb-*infected cotton roots were significantly different from that of V991, showing smaller changes in bacterial diversity and abundance compared to the wild type. These results indicate that GAG polysaccharides play an important role in the interactions between the fungus and rhizosphere soil bacteria. Fungi engage in multiple strategies to manipulate the rhizosphere microbiome. A previous study described that bikaverin, a product of *F. oxysporum*, can act as a "molecular weapon" to manipulate the rhizosphere microbiome, thereby enhancing the pathogen’s pathogenicity [43]. An antimicrobial peptide of *V. dahliae* VdAve1 can inhibit soil bacteria and improve fungal virulence [19]. In our study, treatment with V991 led to increased accumulation of Acidobacteria and Planctomycetota at the phylum level, and decreased the relative abundance of Proteobacteria, Bacteroidota, and Actinobacteria. These phyla are ubiquitous in plant rhizosphere soil and play an important role in plant growth and rhizosphere microbial homeostasis [44]. Most of the bacteria in the Planctomycetota phylum contain enzymes that degrade algal polysaccharides and glycoproteins. Acidobacteria play an important role in the decomposition of soil biopolymers, exopolysaccharide secretion and plant growth promotion [45, 46]. The accumulation of these two phyla in the *ΔVdGtb* strain was less, possibly due to the lack of physical platform or nutrients provided by GAG polysaccharides.

In this study, we found that some bacteria enriched by GAG exhibit strong inhibitory effects against *V. dahliae*. In liquid medium, where direct contact occurs between the fungus and bacteria, *A. animicus* exhibited GAG-dependent inhibitory effects against *V. dahliae*, suggesting that GAG-mediated phycial anchoring is necessary for this specific antagonism. *P. aeruginosa*, whether in liquid or on solid media, significantly inhibited hyphal extension in both wild type and GAG-deficient strains. Although the lack of GAG nearly abolished the binding ability of *A. pittii* to *V. dahliae* hyphae, this bacterium retained comparable inhibitory activity against both strains. This implies that direct physical contact is not the sole determinant of *A. pittii’s* antifungal activity, which likely relies on the secretion of secondary metabolites or on nutrient competition. Although our results suggest that GAG-mediated bacterial recruitment imposes a fitness cost by attracting antagonistic microbes, this interaction must be viewed within the complex ecological context of the plant rhizosphere. GAG may represent an evolutionary trade-off in which the benefits of host adhesion and immune evasion outweigh the risks of attracting certain bacterial competitors. However, it remains unclear how this trade-off plays out in a multi-species bacterial community and whether GAG can recruit beneficial bacteria from the soil.

It was found that *A. pittii*, *A. animicus*, and *P. aeruginosa* were able to induce disease symptoms in plants. However, when these bacteria were co-inoculated with *V. dahliae*, disease symptoms were markedly reduced compared with inoculation with either the bacteria or the fungus alone. This suggests that strong antagonistic interactions occur between the bacteria and *V. dahliae*, resulting in mutual inhibition. A similar phenomenon was observed in GAG-deficient mutants, however, the inhibitory effect was weaker than that observed in the wild-type strain, indicating that GAGs play a crucial role in mediating these interactions. The effects of bacteria on fungal infection are complex. At a low bacterial concentration (OD_600_ = 0.01), *A. pittii* did not cause disease symptoms in cotton but instead enhances fungal virulence in the co-inoculation experiments. These results indicate that intricate interactions exist among phytopathogenic fungi, soil bacteria, plants, and that different bacterial species can exert distinct effects on fungal growth and the infection process.

In summary, GAG polysaccharides located on the fungal surface not only provide protection for the fungi, but also become a platform or target for bacteria to approach the fungi to a certain extent. As a soil dwelling phytopathogenic fungus, *V. dahliae* faces a complex and changing microbial environment and different relationships may exist among them, including competition, mutualism, antagonism, synergism, and so on. Considering the involvement of host plants, more multi-partite interactions may happen. The results present here indicated GAG polysaccharides have important roles in these interactions, the detailed functions warrant further investigation.

## Supplementary Material

GAG_supplementary_materials-ISME-2026509_wrag123

## Data Availability

Raw sequencing data have been deposited in the NCBI Sequence Read Archive (SRA) under the BioProject accession PRJNA1458748, BioSample accessions SAMN57522943–SAMN57522960, and SRA accessions SRR38301218–SRR38301235.
