# Supplementary material for: Galactosaminogalactan orchestrates Verticillium dahliae virulence and rhizosphere microbial ecology through multi-partite interactions
Source: ISME J. 2026 May 17;20(1):wrag123. doi: 10.1093/ismejo/wrag123 (PMC13245733; doi:10.1093/ismejo/wrag123)
Supplement: GAG_supplementary_materials-ISME-2026509_wrag123 [file gag_supplementary_materials-isme-2026509_wrag123.docx]

**Galactosaminogalactan orchestrates *Verticillium dahliae* virulence and rhizosphere microbial ecology through multi-partite interactions**

**Running head:** Multiple roles of galactosaminogalactan.

Xueping Xu ^1#^, Yingqing Tan ^1#^, Sitong Xiecun ^1^, Junyao Wang ^1^, Wennan Zhao ^1^, Lei Wang ^1^, Renyu Dai ^1^, Liting Tang ^1^, Xianbi Li ^1^, Dan Jin ^1^, Yanhua Fan ^1*^.

^1^College of Agronomy and Biotechnology, Southwest University, Chongqing 400715, China.

^#^These authors contributed equally to this work.

^*^Correspondeing author:

Yanhua Fan^*^

College of Agronomy and Biotechnology, Southwest University, No. 2 Tiansheng Road, Beibei District, Chongqing 400715, China.

Email: [fyh@swu.edu.cn](mailto:fyh@swu.edu.cn)

**Supplementary Information**

**Text S1.** Protein accession numbers used for phylogenetic tree construction.

**Text S2.** Analysis of relative expression levels of the *VdGAG* gene cluster.

**Text S3.** Microbiome analysis.

**Text S4.** Interactions between soil bacteria and *V. dahliae*.

**Fig. S1** GAG polysaccharide present in *V. dahliae* conidia.

**Fig. S2** Construction and verification of the *ΔVdGtb* and *VdGtb^com^* vectors in *V. dahliae*.

**Fig. S3** GAG polysaccharides have minimal effects on the growth and development of *V. dahliae*.

**Fig. S4** GAG polysaccharides are required for *V. dahliae* pathogenicity on *Arabidopsis*.

**Fig. S5** GAG polysaccharides mediate attachment of *V. dahliae* to *Arabidopsis* roots.

**Fig. S6** GAG polysaccharides impair the rhizosphere bacterial community of cotton.

**Fig. S7** GAG polysaccharides play important roles in the interactions between *V. dahliae* and bacteria.

**Fig. S8** Relative expression of GAG gene cluster induced by soil and bacteria.

**Fig. S9** Virulence analysis of *V. dahliae* and bacteria in cotton.

**Table S1.** Information of GAG polysaccharides synthesis gene clusters in 16 fungal species.

**Table S2.** Primer sequences used in the article.

**Text S1. Protein accession numbers used for phylogenetic tree construction**

The accession numbers of homologous proteins used for phylogenetic analysis were as follows: XP_009651102.1 (*Verticillium dahliae*), KAG7143257.1 (*Verticillium longisporum*), XP_028498326.1 (*Verticillium nonalfalfae*), TGO62918.1 (*Botryotinia convoluta*), EKD04961.1 (*Trichosporon asahii*), XP_007823134.2 (*Metarhizium robertsii*), XP_016641528.1 (*Scedosporium apiospermum*), KAF3115187.1 (*Orbilia oligospora*), XP_003841319.2 (*Plenodomus lingam*), KAH4268965.1 (*Parastagonospora nodorum*), XP_007704793.1 (*Bipolaris sorokiniana*), KAH1368425.1 (*Aspergillus fumigatus*), GLA48794.1 (*Aspergillus niger*), KAL4768464.1 (*Aspergillus nidulans*), XP_008719224.1 (*Cyphellophora europaea*), XP_002563137.2 *(Penicillium rubens*), APA06882.1 (*Sclerotinia sclerotiorum*), KAH7464054.1 (*Fusarium oxysporum*), and KAJ6087185.1 (*Penicillium canescens*).

**Text S2.** **Analysis of relative expression levels of the *VdGAG* gene cluster**

Analysis of the relative expression of the *VdGAG* gene cluster at different growth stages on solid medium. A conidial suspension of V991 was prepared at a concentration of 1 × 10^7^ conidia / mL. A 100 μL aliquot of this suspension was spread onto solid PDA medium overlaid with a microporous filter membrane and incubated at 26°C. Mycelia were then harvested from three independent plates at 2 and 5 days post-inoculation and used for RNA extraction. Conidia were prepared from 7-day culture using 0.08% Tween-80, and total RNA was extracted. Extracted RNA was reverse-transcribed into cDNA using a reverse transcription kit (Vazyme, China) for subsequent Reverse Transcription‑quantitative PCR (RT-qPCR) analysis. *GAPDH* was used as the internal reference gene. Target genes within the *VdGAG* cluster included *VdUge*, *VdEga*, *VdSph*, *VdAgd*, and *VdGtb*; detailed primer sequences are listed in Table S2.

To analyze the relative expression of the *VdGAG* gene cluster in response to soil and bacterial treatments, a 200 μL conidial suspension of V991 (1 × 10⁷ conidia / mL) was inoculated into 50 mL PDB medium and incubated at 26°C for 5 days. Natural soil (0.5 g) or bacterial cells (0.05 g, wet bacterial pellet) of *A. pittii*, *A. animicus*, or *P. aeruginosa* were then added separately to the cultures, with 500 μL sterile water as a control. After further cultured for 4 h, mycelia were harvested and RNA was extracted. RT-qPCR analysis of the *VdGAG* cluster genes was performed as described above.

**Text S3. Microbiome analysis**

Microbiome analysis were performed by Novogene Bioinformatics Technology Co., Ltd. (Beijing, China) using the QIIME2 pipeline (version 2022.11). Total genomic DNA was isolated from soil samples using the Soil Genomic DNA Extraction Kit (Spin Column Type, TIANGEN). The V4 variable region of the 16S rDNA gene was amplified using primers 515F / 806R (sequences are provided in Table S2). PCR products were purified, pooled equimolarly, and subjected to library preparation with Illumina adapters and barcodes. Sequencing was performed on an Illumina NovaSeq 6000 platform using a paired-end strategy, with a minimum sequencing depth of 50,000 reads per sample and ≥80,000 reads per soil sample. Raw sequencing data (Raw Reads, fastq format) and quality-filtered reads (Clean Reads) were provided by Novogene.

Bioinformatics analyses were carried out using QIIME2. Paired-end reads were processed with DADA2 for quality control and denoising, resulting in the generation of amplicon sequence variants (ASVs). Taxonomic classification was assigned using a naive Bayes classifier trained on the SILVA 138 database. Community diversity was evaluated by calculating alpha-diversity indices (Observed ASVs, Shannon, Simpson, and PD_whole_tree) and beta-diversity dissimilarity matrices (Bray–Curtis, Jaccard, unweighted and weighted UniFrac). Differences in community structure were visualized by principal coordinate analysis (PCoA), statistically assessed using ANOSIM, and Adonis. Differential taxa between groups were identified using LEfSe, DESeq2, and Wilcoxon rank-sum tests. Rarefaction analysis was conducted to confirm sufficient sequencing depth.

**Text S4. Interactions between soil bacteria and *V. dahliae*.**

The bacterial 16S rRNA gene was amplified using the universal primer pair 27F (5’-AGAGTTTGATCCTGGCTCAG-3’) and 1492R (5’-GGTTACCTTGTTACGACTT-3’). The purified PCR products were subjected to Sanger sequencing. The obtained sequences were assembled and blasted against the NCBI nucleotide database to determine the taxonomic affiliation of the tested bacterial isolate. The effect of GAG on bacterial attachment was evaluated by co-culturing bacteria and *V. dahliae* hyphae and quantifying bacteria attached to hyphae. 1 g of hyphae (pre-grown in 100 mL PDB for 3 days) in 5 mL PDB was mixed with 0.5 mL of bacterial suspension (OD_600_  = 0.5) and cultured at 26 °C for 15 h. Bacterial quantification was performed as described above. A *GFP-*expressing *A. pittii* was obtained by transforming the bacterium with the *GFP-*containing *pGFP4412* plasmid and was used to analyze the interactions with *V. dahliae* strains.

The effects of bacteria on fungal growth were determined by liquid co-culture assays and confrontation experiments. In the liquid co-culture experiment, 50 µL bacterial cell suspension (OD_600_ = 0.2) was mixed with 50 µL *V. dahliae* conidial suspension (2×10⁷ conidia / mL). The mixture was inoculated onto CZM and incubated at 26°C for 16 h. Fungal growth was observed microscopically and hyphal length was measured. The growth inhibitory effect was evaluated by calculating the ratio of hyphal length in the cocultured group to that in the control group. For confrontation assays, 2 µL of a bacterial suspension (OD_600_ = 0.1) and conidial suspension (1×10⁷ conidia / mL) were spotted onto the same PDA plate at a distance of 1 cm apart. Plates were incubated at 26°C for 3 days and fungal growth inhibition was observed.


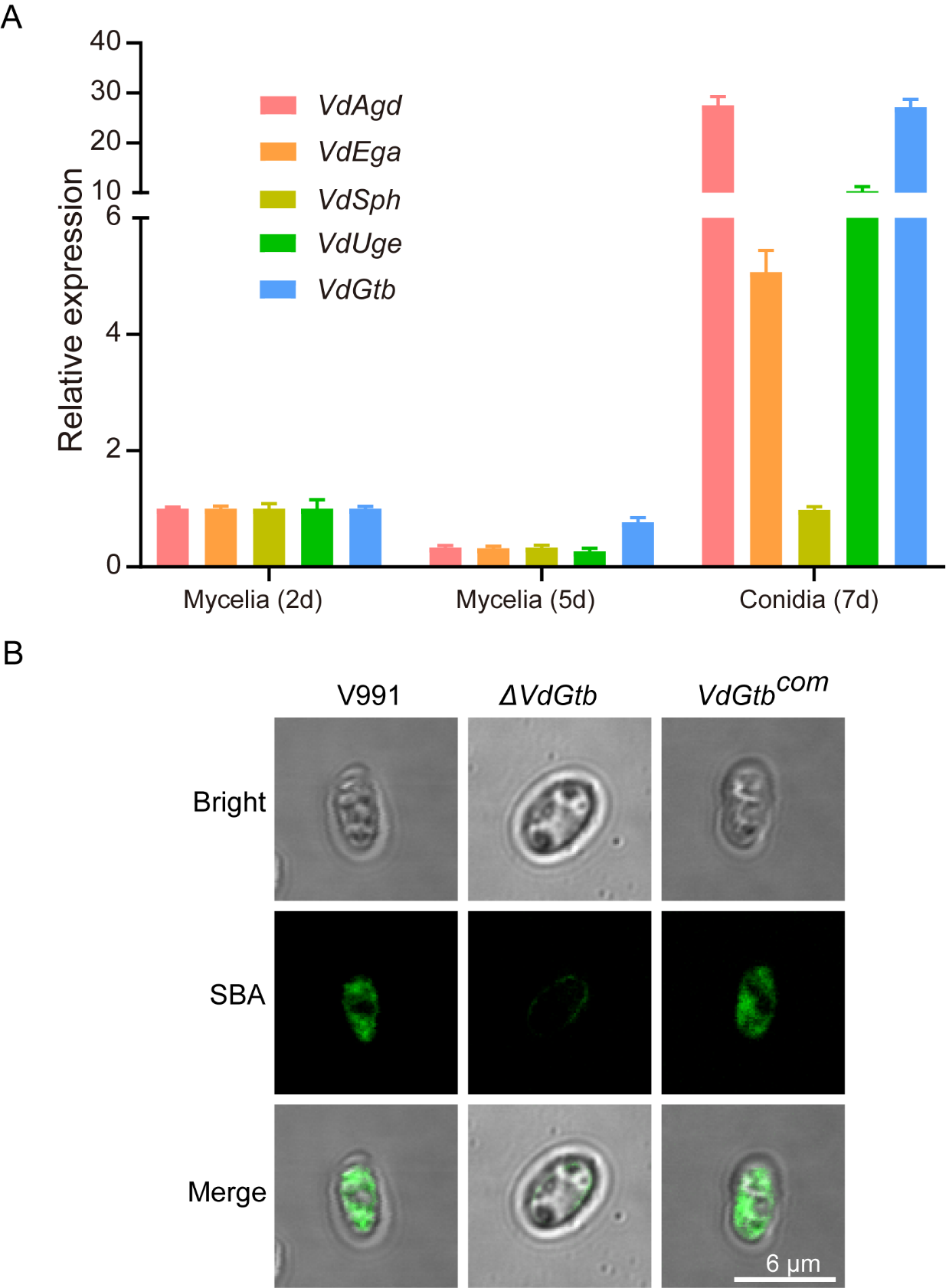


**Fig. S1 Detection of GAG polysaccharides synthesis in *V. dahliae*. A** Detection of the relative expression levels of the *VdGAG* gene cluster during *V. dahliae* growth and development. *V. dahliae* was cultured on PDA medium for 2 and 5 days to obtain mycelia. Conidia were collected from 7-days PDA plates. **B** SBA staining of GAG polysaccharides in conidia. Conidia were harvested from various strains cultured on solid PDA medium for 7 days. Scale bar, 6 μm.


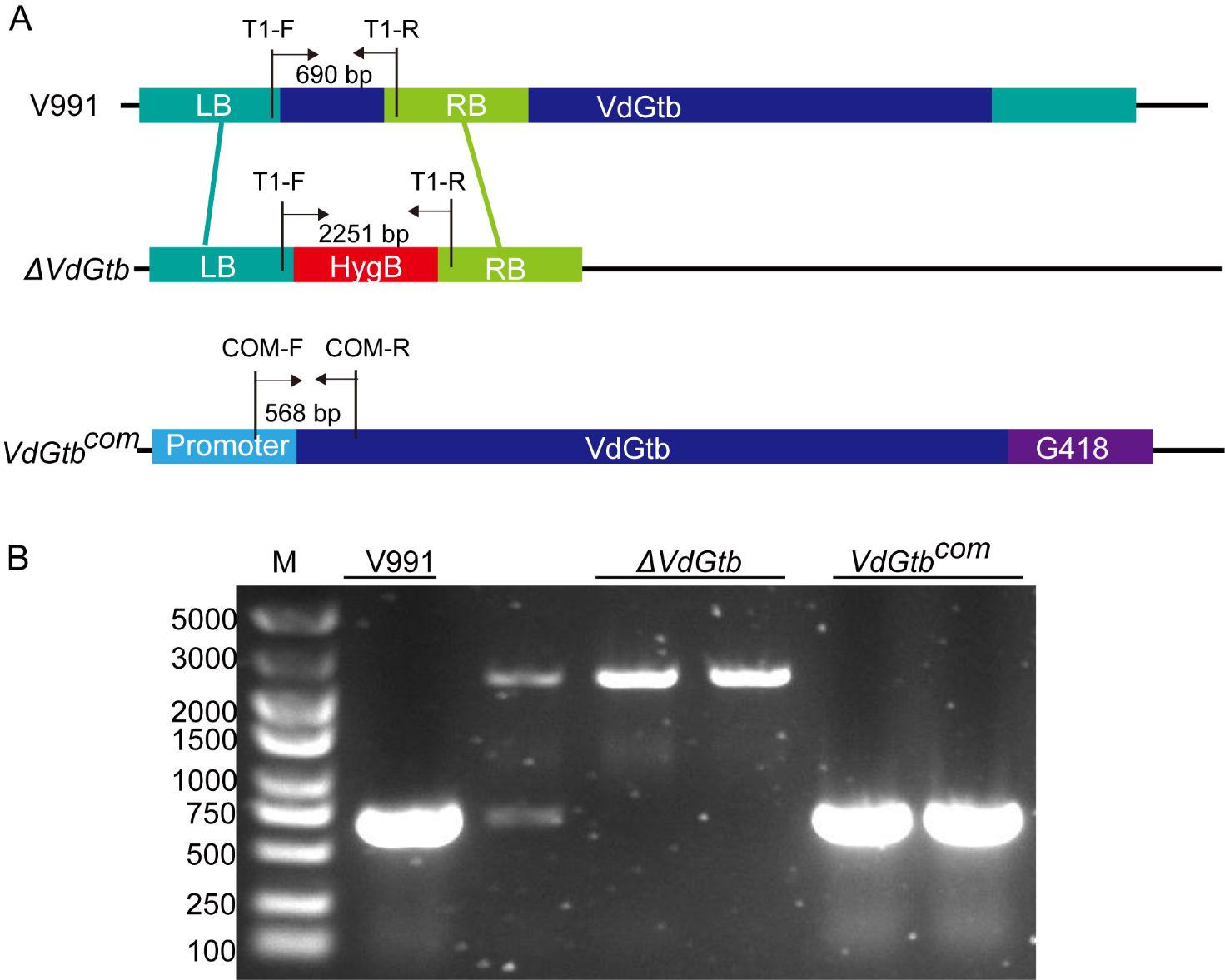


**Fig. S2 Construction and verification of the *ΔVdGtb* and *VdGtb^com^* vectors in *V. dahliae*. A** Schematic diagrams of vector constructs. The hygromycin resistance gene and geneticin resistance gene were used for mutant and complemented strains screening. Primers (T1-F / R and COM-F / R) were used for PCR detection. **B** Verification of transformants by PCR. M, DNA marker (bp). The PCR products (with primersT1-F / R) from V991 and *ΔVdGtb* were 690 bp and 2251 bp, respectively. The PCR product from *VdGtb^com^* (with primers COM-F / R) was 568 bp.


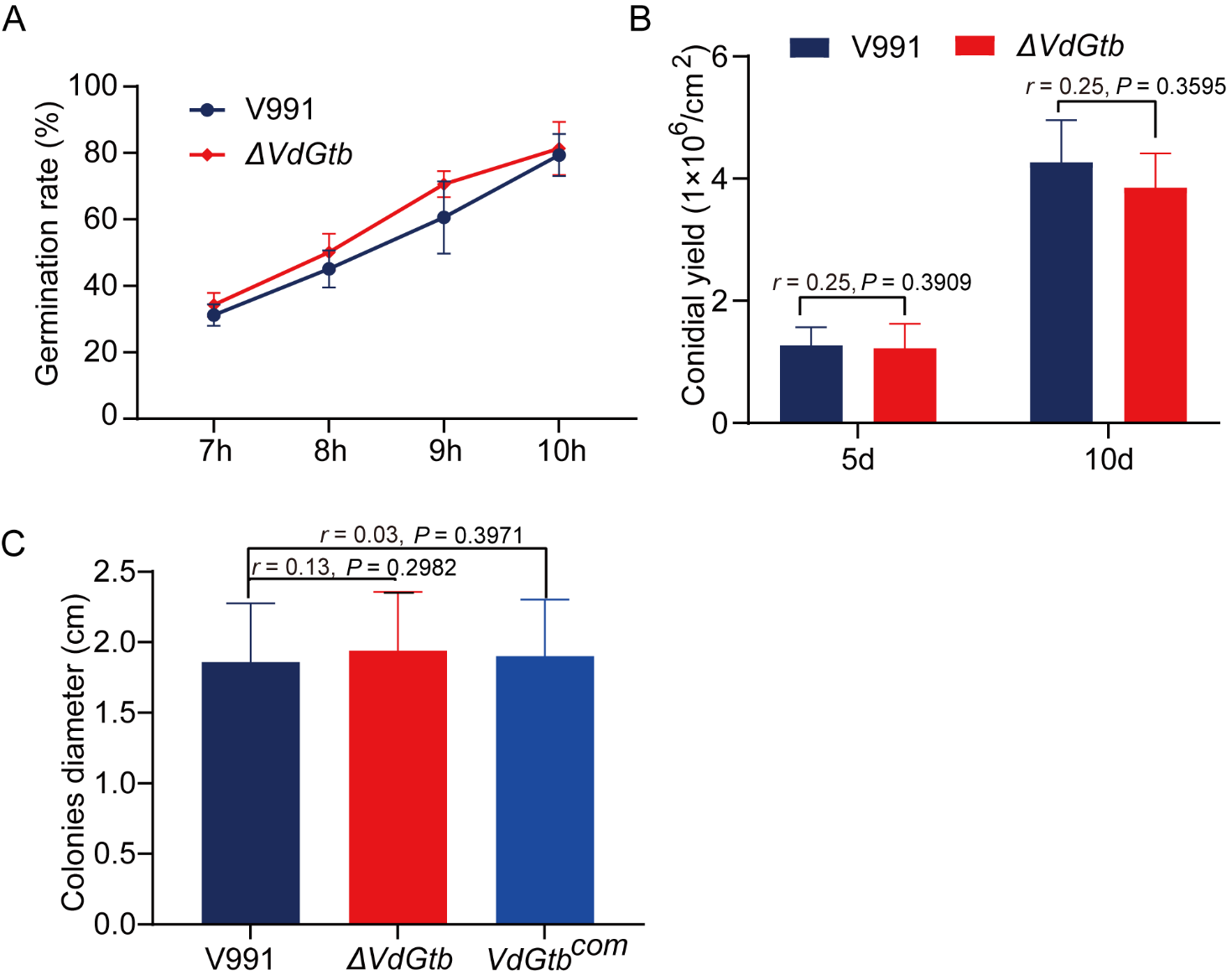


**Fig. S3 GAG polysaccharides have minimal effects on the growth and development of *V. dahliae*.** **A** Conidial germination rate on CZA medium at different points in time. **B** Conidial yields of V991 and *ΔVdGtb*. Fungi were cultured on CZA for 5-10 days. **C** Penetration analysis. Colonies were observed after fungal penetration of cellophane on CZA medium. Plotted data represent the mean ± SEM. Data were normally distributed (Shapiro-Wilk test, *P* > 0.05) and analyzed using an unpaired *t*-test. n≥3.


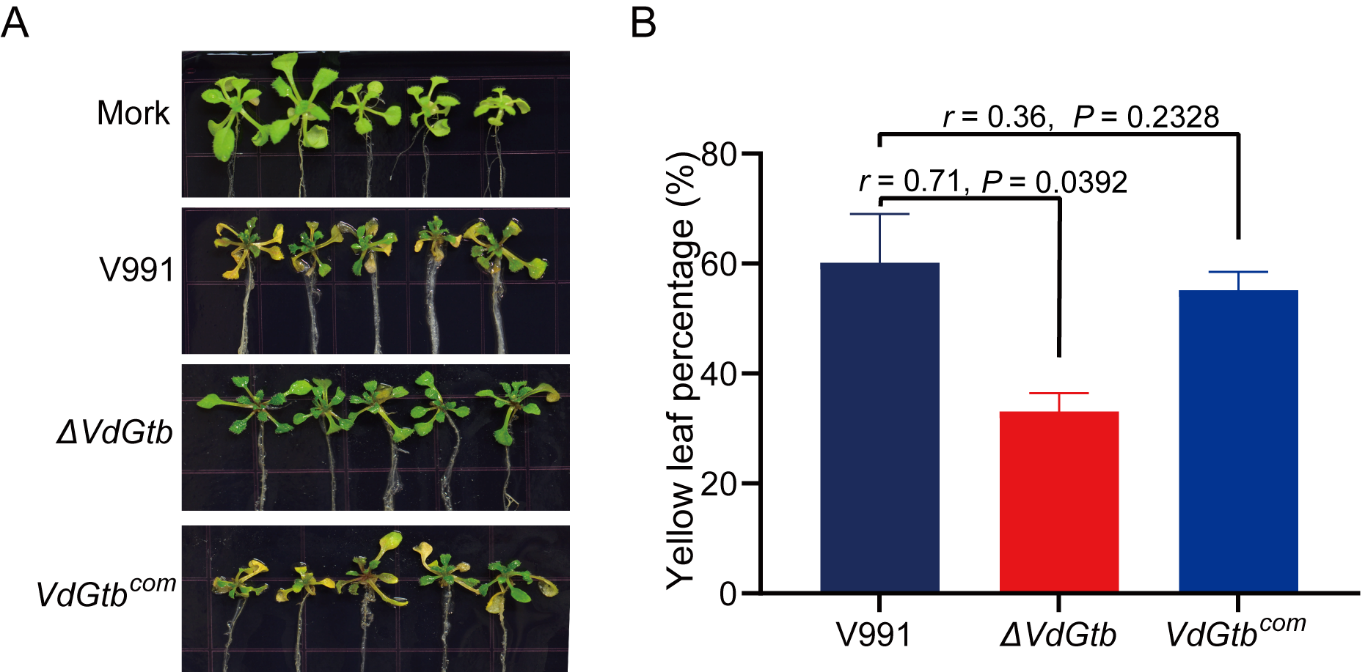


**Fig. S4 GAG polysaccharides are required for *V. dahliae* pathogenicity on *Arabidopsis*. A** Disease symptoms of *Arabidopsis* plants inoculated with various fungal strains after 10 days. **B** Quantification of the percentage of yellow leaves from the plants shown in Fig. S4A. Plotted data represent the mean ± SEM. Data were normally distributed (Shapiro-Wilk test, *P* > 0.05) and analyzed using one-way ANOVA followed by Tukey’s multiple comparisons test. n ≥ 3.


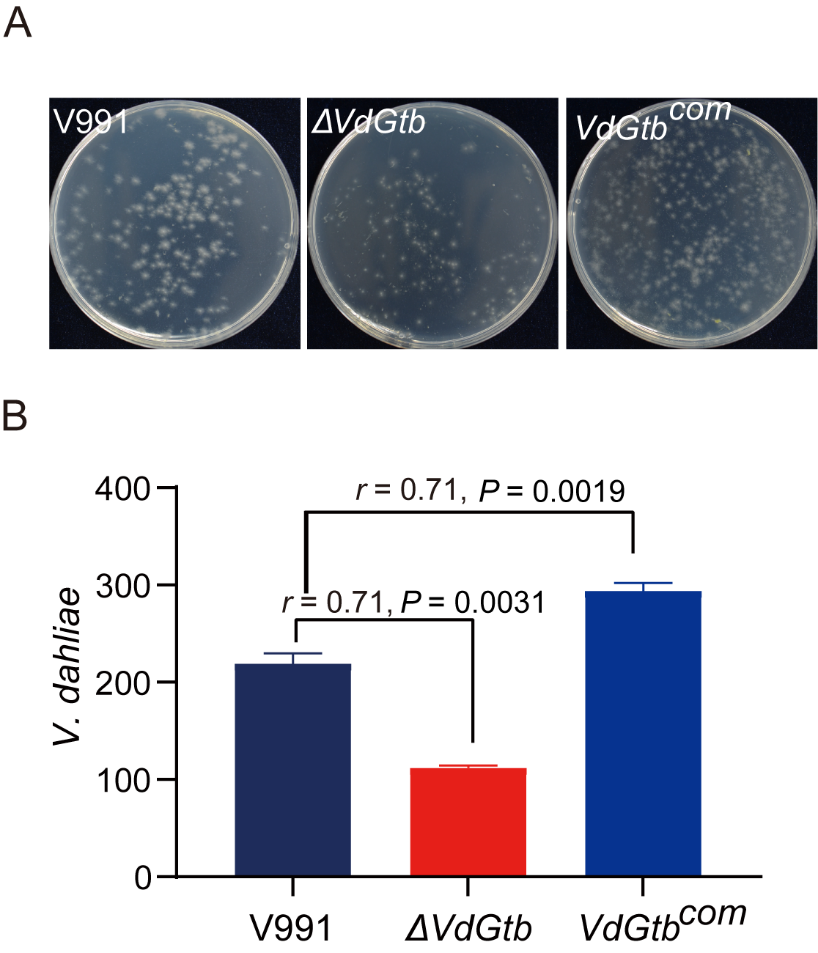


**Fig. S5 GAG polysaccharides mediate attachment of *V. dahliae* to *Arabidopsis* roots. A** Representative images of fungal colonies attached to *Arabidopsis* roots. Fungal conidia were inoculated onto *Arabidopsis* roots and isolated from the roots 15 h post inoculation. **B** Quantification of *V. dahliae* attachment to *Arabidopsis* roots from Fig. S5A. Plotted data represent the mean ± SEM. Data were normally distributed (Shapiro-Wilk test, *P* > 0.05) and analyzed using one-way ANOVA followed by Tukey’s multiple comparisons test. n≥3.


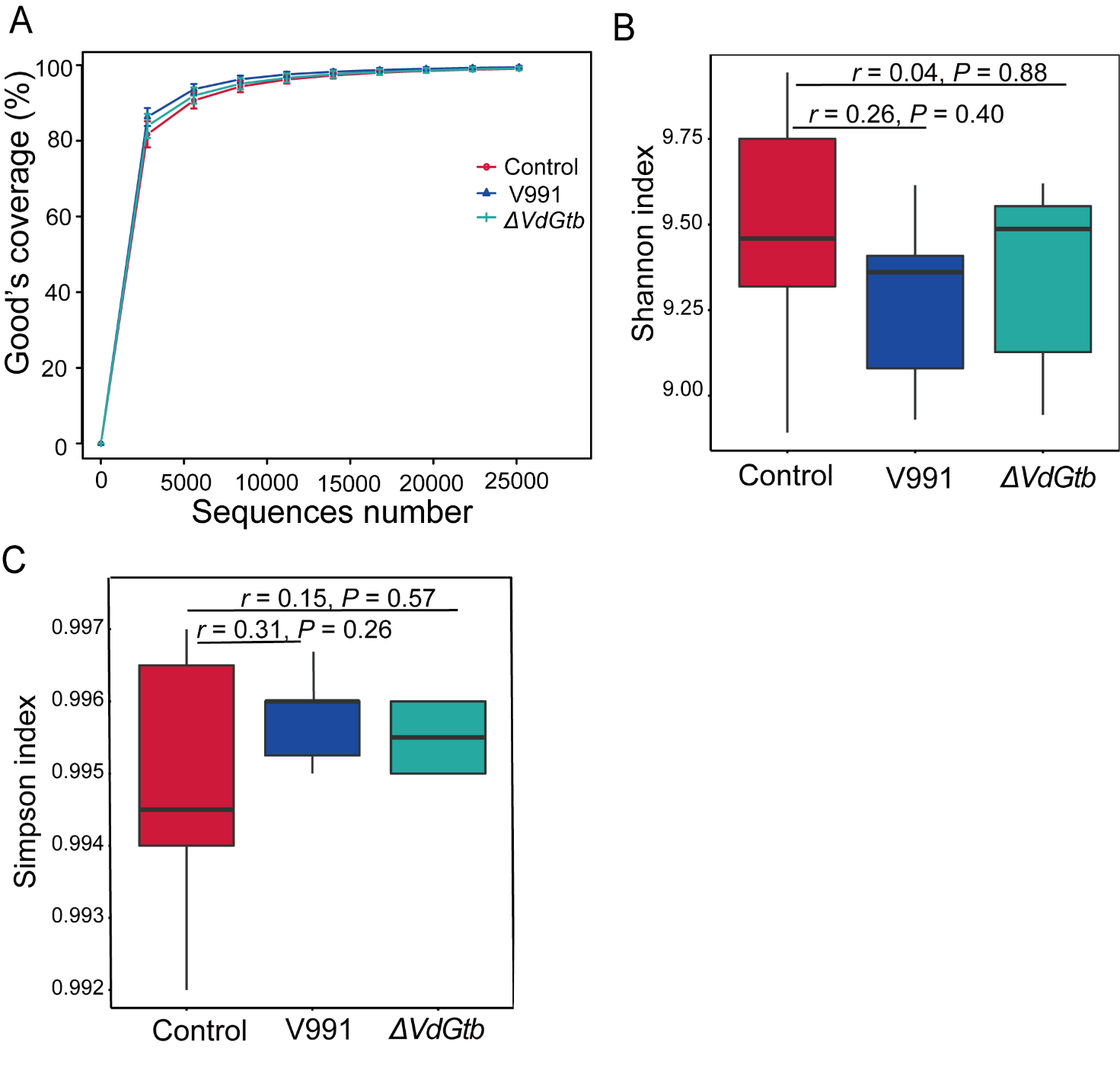


**Fig. S6 GAG polysaccharides impair the rhizosphere bacterial community of cotton. A** Good’s coverage index for all root soil groups (Control, V991, *ΔVdGtb*). **B** Alpha-diversity index (Observed features) of the bacterial community. **C** Shannon index of the rhizosphere bacterial community. **D** Simpson index of the rhizosphere bacterial community. Data are presented as mean ± SEM. Kruskal–Wallis test, each treatment included six biologically independent replicates (n = 6).


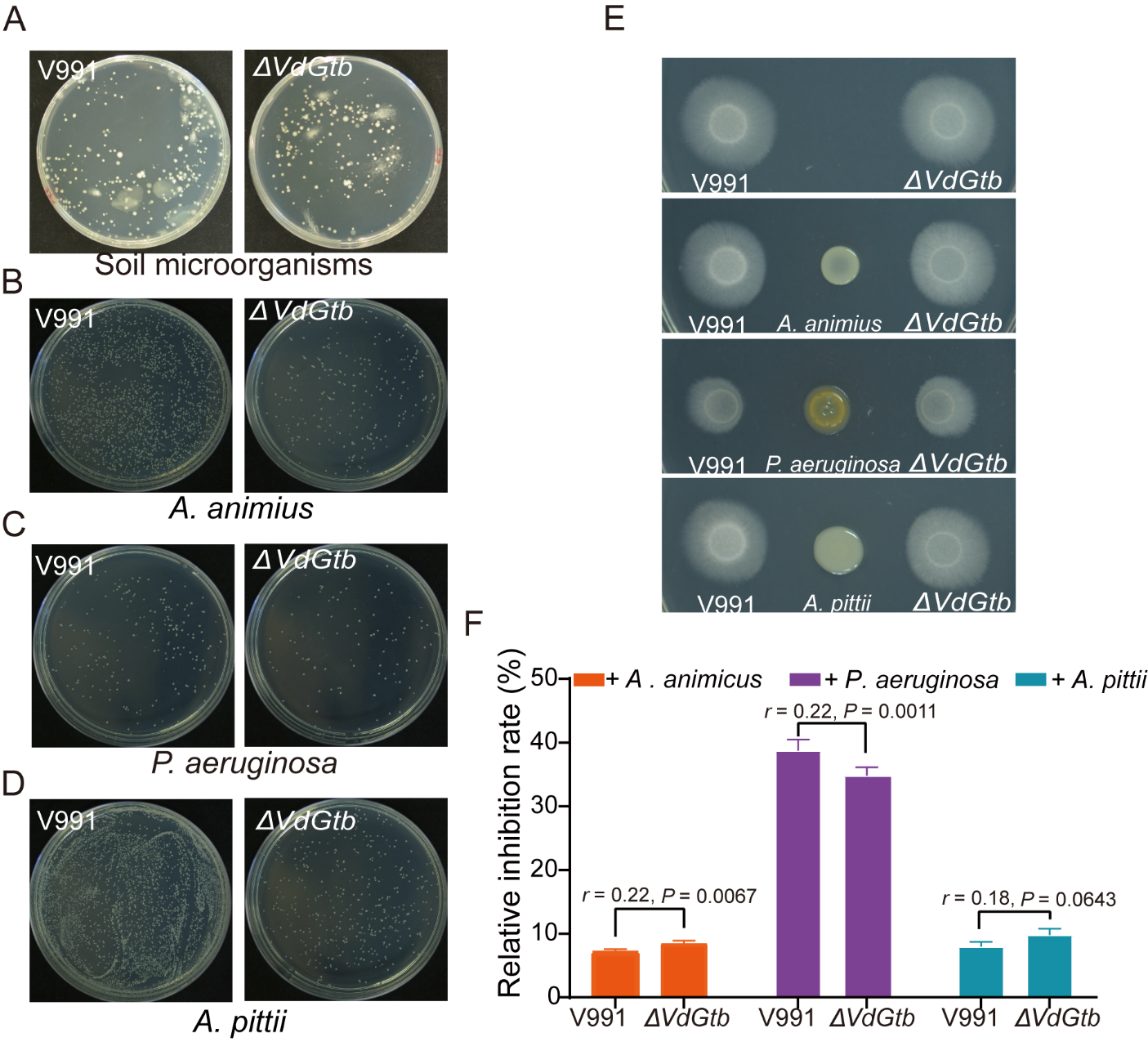


**Fig. S7 GAG polysaccharides play important roles in the interactions between *V. dahliae* and bacteria.** **A-D** Bacteria enriched on fungal hyphae. Culturable bacterial colonies were re-isolated from fungal hyphae after co-culture with soil microorganisms (**A**), *A. animicus* (**B**), *P. aeruginosa* (**C**), and *A. pittii* (**D**). **E** Colony confrontation assay. Fungi and bacteria were cultured on PDA for 3 days. **F** Quantification of the relative inhibition shown in Fig. S7E. Data were represented as the mean ± SEM. Data were normally distributed (Shapiro-Wilk test, P > 0.05) and analyzed using one-way ANOVA followed by Tukey’s multiple comparisons test. n≥3.


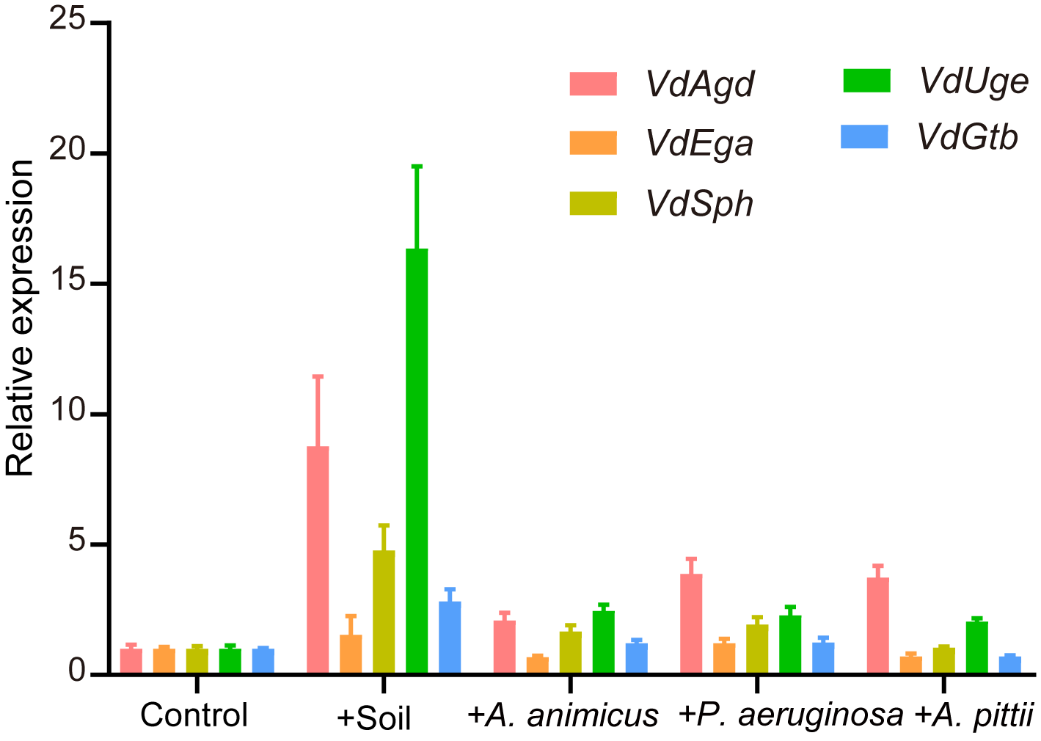


**Fig S8 Relative expression of GAG gene cluster induced by soil and bacteria.** Control: Fungal mycelium cultured in PDB liquid for 5 days; +Soil: Soil sample added; +*A. animicus*, +*P. aeruginosa*, +*A. pittii* are experimental groups with the corresponding bacteria added. To highlight the differences, the relative expression of genes within the *VdGAG* gene cluster in the Control group was set to 1.


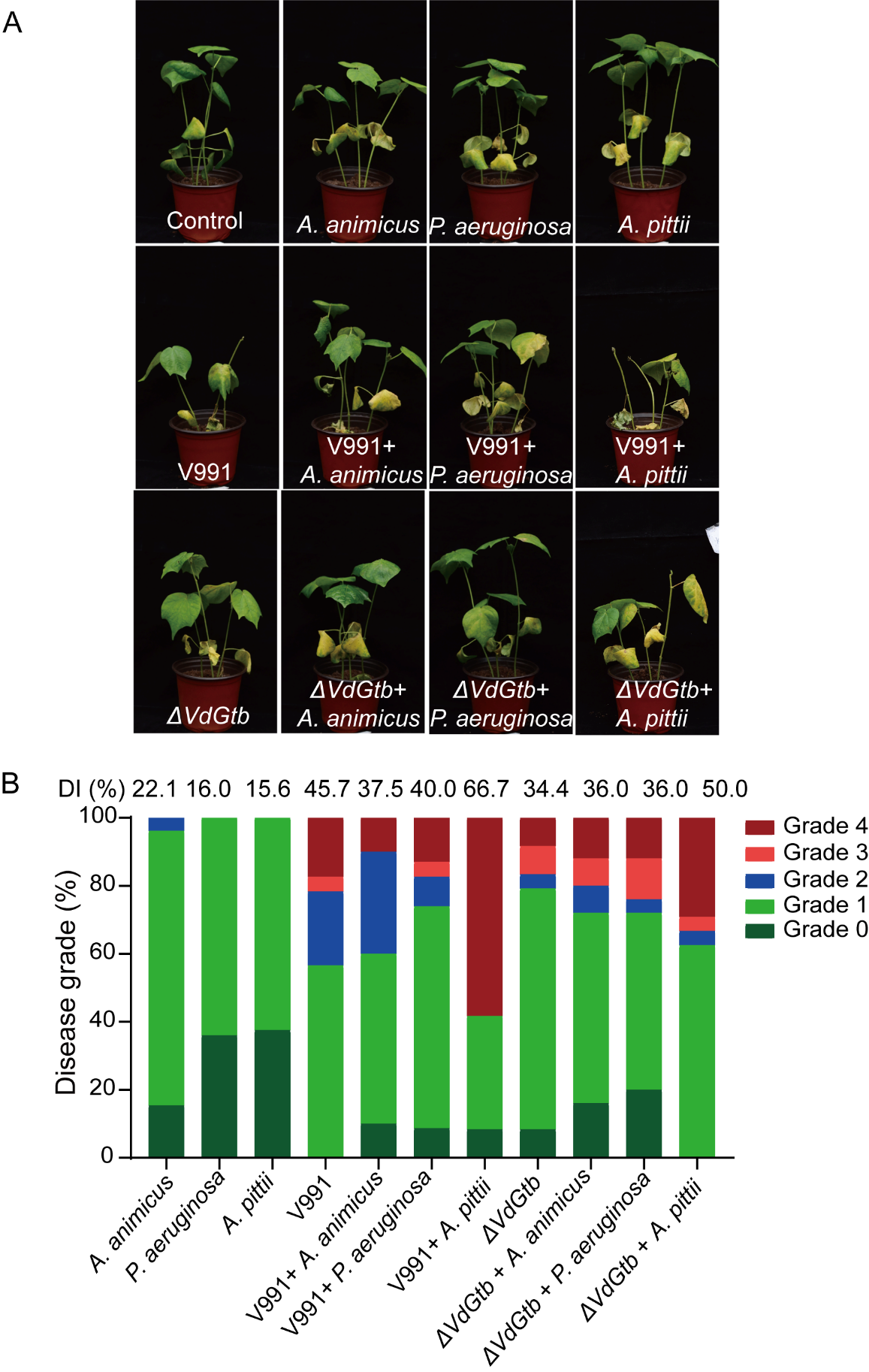


**Fig. S9 Virulence analysis of *V. dahliae* and bacteria in cotton. A** Disease symptoms of cotton inoculated with V991 or *ΔVdGtb* alone or in combination with bacteira (each at OD_600_ = 0.01) after 25 days. **B** Disease index and grade distribution for the treatments shown in Fig. S9A. n≥3.

| Fungal species | GAG biosynthetic gene cluster | | | | |
| --- | --- | --- | --- | --- | --- |
| *A. fumigatus* | Afu3g07870 (agd3) | Afu3g07890 (ega3) | Afu3g07900 (sph3) | Afu3g07910 (uge3) | Afu3g07860 (gtb3) |
| *M.robertsii* | MAA_06941 (MrAgd;48%) | MAA_06942 (MrEga;47%) | MAA_06943 (MrSph;34%) | MAA_06944 (MrUge;51%) | MAA_06945 (MrGtb;28%) |
| *V.dahliae* | VDAG_08037 (Vdagd;49%) | VDAG_08038 (VdEga;51%) | VDAG_08039 (VdSph;34%) | VDAG_08040 (VdUge;54%) | VDAG_08041 (VdGtb;33%) |
| *A.nidulans* | AN2954 | AN2953 | AN2952 | AN2951 | AN2955 |
| *M.anisopliae* | MAN_07185 | MAN_07186 | MAN_07187 | MAN_07188 | MAN_07189 |
| *M.brennum* | MBR_09142 | MBR_09141 | MBR_09140 | MBR_09139 | MBR_09138 |
| *M.guizhouense* | MGU_09803 | MGu_09802 | MGu_09801 | MGU_09800 | MGu_09799 |
| *M.majus* | MA_08446 | MAl_08447 | MAJ_08448 | MA_08449 | MA_08450 |
| *M.rileyi* | NOR_03856 | NOR_03857 | NOR_03858 | NOR_03859 | NOR_03860 |
| *M.acridum* | MAC_02143 | MAC_02142 | MAC_02141 | MAC_02140 | MAc_02139 |
| *M.album* | MAM_08305 | MAM_O8304 | MAM_08303 | MAM_08302 | MAM_08301 |
| *N. crassa* | NCUO5137 | NCUO5136 | NCUo5135 | NCUO5133 | NCUO5132 |
| *N. tetrasperma* | xp_009854704 | xp_009854705 | xp_009854706 | xp_009854708 | xp_009854709 |
| *S.sclerotiorum* | ssiG_04473 | ssIG_04475 | ssiG_04476 | ssiG_04477 | sSIG_04472 |
| *B. cinerea* | BC1G_00448 | BCIG_00447 | BCIG_00446 | BCIG_00445 | BCIG_00451 |
| *A.oligaspora* | xp_011120260 | xp_011123749 | xp_011119552 | xp_011117653 | xp_011119554 |

**Table S1. Information of GAG polysaccharides synthesis gene clusters in 16 fungal species.**

**Table S2. Primer sequences used in the article.**

| Primer name | Sequence（5’-3’） | Use |
| --- | --- | --- |
| LB-F / R | AGCTATGACCATGATTACGAATTCTGCCGAAGGGGGTATTCCTA  TCTTCTGTCGACACTAGTGAATTCTCGTGAAAGGGGGTTTGAGG | Amplification of the *VdGtb* homologous left arm. |
| RB-F / R | GTCGACCTGCAGGCATGCAAGCTTCTCCCATGGGCCATCTTGTG  AAAACGACGGCCAGTGCCAAGCTTGAAGTCAGTCGGTGCTCAGG | Amplify the right arm of the *VdGtb* homolog. |
| T_1_-F / R | CTCAAACCCCCTTTCACGA  GTCAGTAACCAGAACCTAG | Screening for *VdGtb* knockout mutants. |
| PG-F / R | GTAAAACGACGGCCAGTGCCAAGCTTCTTTGTTTGCTCGTTGTCTGAG  CTAGAACTAGTGATATCAAGCTTTTATGCCGGGCTAACGCTTGTGCG | Amplification of the promoter and *VdGtb* gene fragments. |
| COM-F / R | CCTACATCACCTCAAACCCC  CTACTCGGTAGAGGAGGAAG | Screening for *VdGtb*-complementing strains. |
| VdGtb-F / R | GCCATTGTCATCCCCATCCT  TGGGAGAGGACCCATCTCTG | RT - qPCR detection of the *VdGtb* gene. |
| VdActin-F / R | GTCCATCTTGCCCTCTTTCCA  TCCCTCACCTTCCTTCGGAT | *Verticillium dahliae*  RT-qPCR internal reference gene. |
| GhHIS3-F / R | CCGTCCTGGAACTGTTGCTCT  ACCCACAAGGTATGCCTCTGC | Cotton RT -qPCR internal reference gene |
| GHPR1-F / R | GGGGCAGTGCTGACCTATCG  TTAGCACAACCAAGATGGACAGAGT | Detection of cotton-related immune genes. |
| GHPR2-F / R | CTCCGCTTTCGCCTATTCCA  CGCACAGATGACGCTGATGA |  |
| GHPR3-F / R | GATGACTCCACAATCACCGAAGC  GCGGTCTTCTACCTGGGCATT |  |
| GHPR5-F / R | ATTTGACAACTCTGGCTCTGGGA  GTAAAAGTCCTTATCCCCCGAGC |  |
| GHICSI-F / R | GTCTTCAGCCACCTAATGGACCCGC  GCTCTGGATTCACCTCTAGCACG |  |
| VdAgd-RT-F / R | CGTCCTTCTGGCTCGAGTTT  CAAGAGGAGTGGCAGGGAAG | Detecting the relative expression levels of the GAG gene cluster. |
| VdEga-RT-F / R | GAACAACAACGACAACGGCA  ATGAGCACAATCTGCCACGA |  |
| VdSph-RT-F / R | CATATGCTACCTCGGCCTCG  TGCCTTTGCTCCACTTCGTA |  |
| VdUge-RT-F / R | CACTTTGCGGCGTTCAAGTC  AAATGTTGTGCTTGCCGAGG |  |
| 515F / 806R | GTGCCAGCMGCCGCGGTAA  GGACTACNNGGGTATCTAAT | Amplification of the V4 Region of 16S rDNA. |
